# The impact of hair coat color on longevity of Holstein cows in the tropics

**DOI:** 10.1186/s40781-016-0123-3

**Published:** 2016-12-12

**Authors:** C. N. Lee, K. S. Baek, A. Parkhurst

**Affiliations:** 1 Department of Human Nutrition, Food and Animal Sciences, University of Hawaii-Manoa, Honolulu, HI 96822 USA; 2 National Institute of Animal Science, RDA, Jeju Island, South Korea; 3 Department of Statistics, University of Nebraska-Lincoln, Lincoln, NE 68583 USA

**Keywords:** Hair coat color, Survival, Milk production, Longevity, Holstein cows, Tropics

## Abstract

**Background:**

Over two decades of observations in the field in South East Asia and Hawai‘i suggest that majority of the commercial dairy herds are of black hair coat. Hence a simple study to determine the accuracy of the observation was conducted with two large dairy herds in Hawaii in the mid-1990s.

**Methods:**

A retrospective study on longevity of Holstein cattle in the tropics was conducted using DairyComp-305 lactation information coupled with phenotypic evaluation of hair coat color in two large dairy farms. Cows were classified into 3 groups: a) black (B, >90%); b) black/white (BW, 50:50) and c) white (W, >90%). Cows with other hair coat distribution were excluded from the study. In farm A, 211 out of 970 cows were identified having 4 or more lactations. In farm B, 690 out of 1,350 cows were identified with 2 or more lactations for the study.

**Results:**

The regression analyses and the Wilcoxon-Log-rank test for survival probability showed that Holstein cattle with 90% black hair coat had greater longevity compared to Holstein cattle with 90% white hair coat.

**Conclusions:**

This study suggests that longevity of Holstein cattle in tropical regions was influenced by hair coat color and characteristics.

## Background

The effect of hair coat on beef cattle production has been reported in several studies Yeates [33], Turner and Schleger [27], Finch et al. [7], and Gilbert and Bailey [12].

Turner and Schleger [27] reported that for each one point increase in hair coat thickness, there was an estimated 11.2 kg loss in average daily gain. The role of coat color in cattle was further highlighted by Finch et al. [7] who reported significant effect of color (dark vs light) and hair coat thickness (wooly) negatively impacted grazing time and growth. Gilbert and Bailey [12] did not find any influence in hair coat characteristics on post-weaning gain for the population of Angus and Hereford in a Canadian feedlot.

King et al. [18] suggested that white coat Holstein freshening in February and March had lower days open and required fewer services per conception. However, the higher milk production observed in white cows was attributed to the influence of sire. Lower milk production was reported for Holsteins cows with black hair coat vs white hair coat (3.3 kg. vs 1.5 kg.) in no shade environment [14]. The number of animals in this study was small (9 white and 11 black cows) and the author stressed the need for further evaluation of hair coat and heat stress. Work in the US Virgin Islands, tropical climate with hot-humid weather showed that the majority of the Holstein cows in that study had black hair coat color [13]. The milk production was not statistically different between black vs. white hair coat cows in that study.

Recent changes in climate [4] suggest that the interactions between hair coat and hair color in livestock needs to be reexamined. Increased frequency of heat waves have been noted in the past decade [5] and the more recent historic heat wave of 2013 [25] warrants a better understanding of animals survival under such circumstances. In an earlier study with beef cattle, Frisch [8] reported that it was possible to select for animals with greater heat tolerance. Hence, the objectives of this study are to: a) evaluate the production (milk yield per lactation) of Holstein cattle in the tropics as related to hair coat color, and b) determine if there is a relationship between hair coat and longevity for Holstein herds in the tropics. Longevity is important in dairy cattle when the cost of raising heifers from birth to first lactation can be astronomical in places where land prices are high and most of the feed are imported; e.g. Hawai’i.

## Methods

Data from two large commercial dairies in the Waianae district, within <0.25 miles apart, were used for the study. Farm A milked 950–1000 head of Holsteins while farm B milked 1,350 –1450 head Holsteins cows. Farm A employed fans with misters targeted at the loafing area and sprinklers at the feed manger to cool cows. Farm B had sprinklers at the feed manger with fans overhead and fans and sprinklers in the holding barn of the milking parlor. Both farms were open lot operations with corrugated roof for shade at 12–14 ft above the loafing area. They feed a total mixed ration; Farm A 4x per day and Farm B 2x per day. Dairy-Comp 305 software was employed in both farms for data keeping.

Cows that were included in the study had to meet the following criteria: a) minimum of 4 lactations in farm A and min. of 2 lactation for farm B, b) the body hair coat color had to be either 90% black or 70% white or 50:50 (black:white) by visual evaluation and c) had complete 305d lactation records. The minimum lactation set for the respective farms were decided base on the number of animals that can be identified with increasing lactation numbers. Farm B had less aged cows with 5 or more lactations (*n* = 56). Farm A had 114 cows with 5 or more lactations. This criteria on lactation ensured that there would be a minimum 30 cows for each lactation group in a farm.

Dairy Comp 305 database was used to generate the list of animals that met the milk and lactation criteria. Armed with the list, two individuals would performed independent visual evaluation of hair coat at each farm. They identified the color of the cows and the pens for the respective farms. Then the list was crossed checked. If there was any disagreement, the individuals would revisit the pen(s), reevaluate the cow and come to an agreement on the hair coat color. If they should failing to come to an agreement on the hair coat color, that cow would be excluded from the study.

Based on the complete list, the following analyses were done: a) distribution of animals by hair coat for the respective farms, b) evaluation of milk production by hair coat (multivariate analyses of variance), c) regression analyses by lactation number and d) further evaluation of data by Wilcoxon and Log-rank test for homogeneity.

In addition, samples of hair between the black and white animals (*n* = 11 cows for each group were randomly selected from cow ID draw) were clipped using a Wahl® Arco cordless clipper at the 11^th^ rib region and evaluated for weight (μg/cm^2^).

## Results and discussion

### Population distribution by hair coat

In Farm A, 211 animals met the criteria for the study while in Farm B, 690 cows were identified. The distribution of these animals by hair coat color was presented in Table 1. Majority of the animals in Farm A that had 4 or more lactations were black (59.7%), B:W (28.0%) and W (12.3%). In Farm B, the cow distribution by hair coat color were B (47.4%), B:W (39.3%) and W (13.3%). Cows with 70% or more white hair coat formed the smallest population; Farm A - 12.3% and Farm B - 13.3%. A greater population of black cows was reported for the US Virgin Island [13]. In earlier work with goats, Finch et al. [6] concluded the additional heat load in black goats was lost by higher rate of evaporative cooling. Greater evaporative heat loss by black Holstein cows (90% black) over white Holstein cows (90% white) was observed by Hillman et al. [17]. The sweating is 1.6x higher for black cows [17]. Similarly, Finch et al. [6] reported 1.4x higher sweating rate for black goats. Both studies reported higher skin temperature in the black animals. Findings by Gebremedhin et al. [11] showed hair coat had an influence on sweating rate in Holstein cows. For the white unshaved cows, the sweating rates were only 87% of that measured in unshaved black cows. No differences were observed when the animals were in shaded environment. It was suggested that there was an inverse relationship between sweating rates and skin temperature which consequently increased the cooling rate at the skin level.

**Table 1:** Distribution of cows by hair coat: black, white and black and white in the farms

Farm ID	Number of cows	Hair coat distribution (%)		
		Black (>90%)	Black:white (%; 50:50)	White (>90%)
Farm A	211	59.7^a^	28.0^b^	12.3^c^
Farm B	690	47.4^a^	39.3^b^	13.3^c^

Different superscript within a column denotes *P* < 0.05

### Milk production

Table 2 shows the milk production by hair coat for both Farm A and Farm B. In both farms, white cows had higher milk production over black cows but this was not statistically different. The higher milk production observed in Holsteins with white hair coat for the first and second lactations by Becerril et al. [2] and for the first lactation by Maia et al. [19]. Hansen [14] reported a greater depression in milk yield for black cows (3.3 kg.) in no shade environment over white cows (1.5 kg.) and attributed this to the higher body temperatures for the black animals. He further indicated that it was not clear if it would be useful to make selection based on hair coat color even though white hair coat is fairly heritable, (>0.7; [3]). A study in Arizona by Rundle [26] concluded that first lactation production was not related to or not influenced by hair coat color. King et al. [18] suggested that white cows did not have significantly higher 305d ME in milk production then mixed or black cows. Both these two studies supported the current findings. Sires predicted differences for production traits had a greater influence in milk yield [18]. In King et al. [18] study, there seem to be a shorter calving interval to conception (days) for white cows for the period of Feb. to Mar. However, black cows seem to have advantage for the period of Aug.–Sept., the hotter months. If one were to examine the data further, the combined annual data would yield no advantages in these reproductive parameters by hair coat. Rundle [26] and Godfrey and Hansen [13] are in agreement about the lack of clear advantage of hair coat color on reproductive traits. In our current study we did not perform any direct measures on reproductive traits. If one accepts that milk production is a by-product of successful reproduction, than one can infer that the higher percentages of cows with black hair coat in the tropics suggested that their reproduction was not hampered; otherwise they would have been culled for low production or failure to reproduce. Physiologically, the greater efficiency in evaporative cooling in black cows [11, 17] in cows and Finch et al. [6] in goats may abated any detrimental effect of higher day time body temperatures. The dynamics of plumage color and skin interface under solar was well described by Walsberg [30] who showed that radiation penetrates deeply in white plumage pigeons. Gebremedhin and Hillman [10] showed that the peak temperature in black fur was on the outer surface while the peak temperature for white fur was deeper in the hair coat. Similarly, Maia et al. [20] demonstrated that white hair follicle had more effective thermal conductivity. This phenomena could post harmful effects for Holstein with white hair coat as the skin under such coat is pink (Figure 4).

**Table 2:** Milk production (kg./305d lactation; mean ± SE)

Farm ID	Number of cows	Black	Black: white	White
Farm A	211	11,535.0 ± 253	11,121.0 ± 330	11,830 ± 187
Farm B	690	9,612.7 ± 207	9,920.0 ± 234	9,928 ± 214

### Longevity determination by regression analyses

**Fig. 1:**
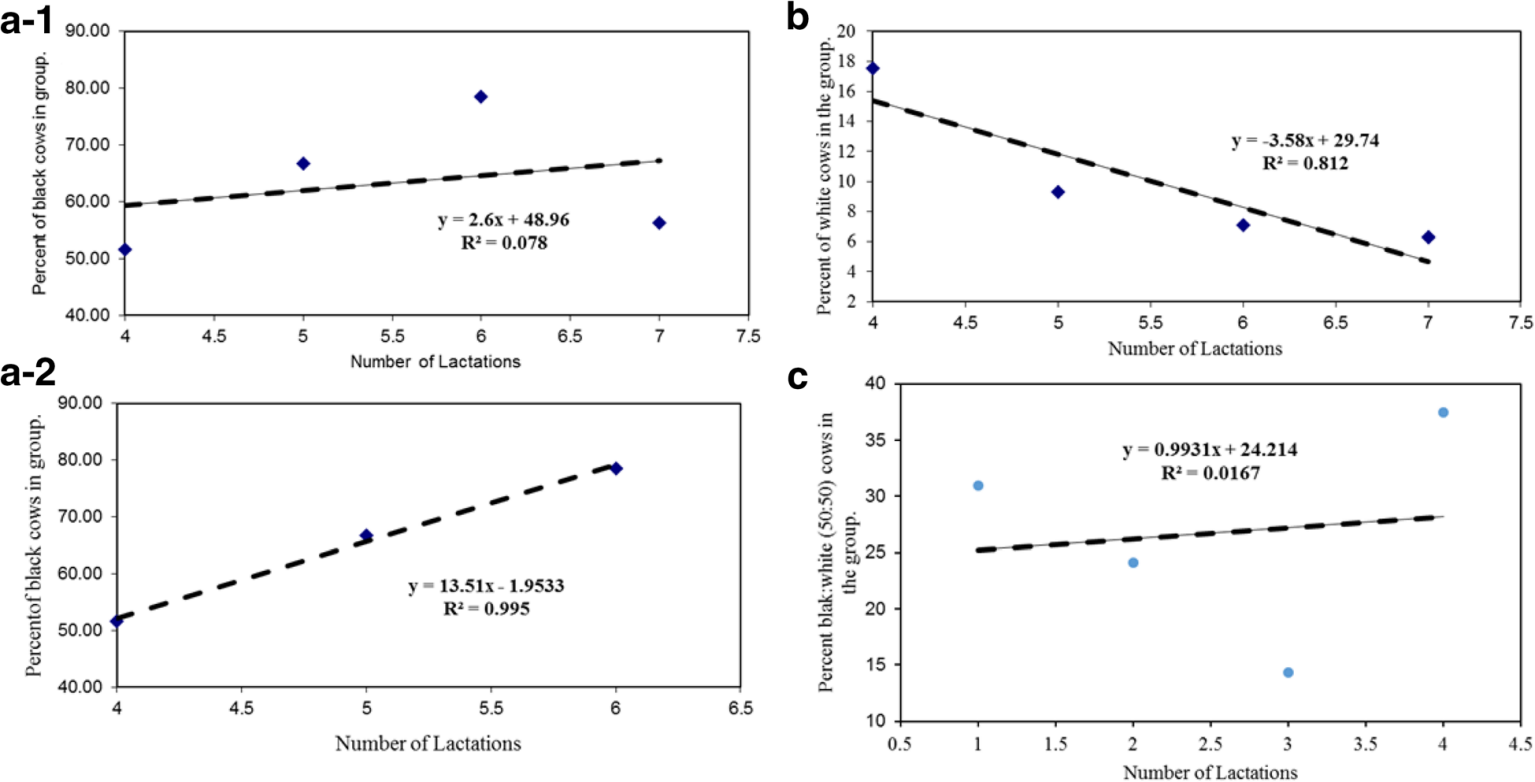
**a**-1 Regression analyses of black hair coat cows with 4 to 7 or more lactations for Farm A. **a**-2 Regression analyses of black hair coat cows with 4 to 6 lactations for Farm A. **b** Regression analyses of white hair coat cows with 4 to 7 or more lactations for Farm A. **c** Regression analyses of black:white (50:50) cows with 4 or more lactations in Farm A

**Fig. 2:**
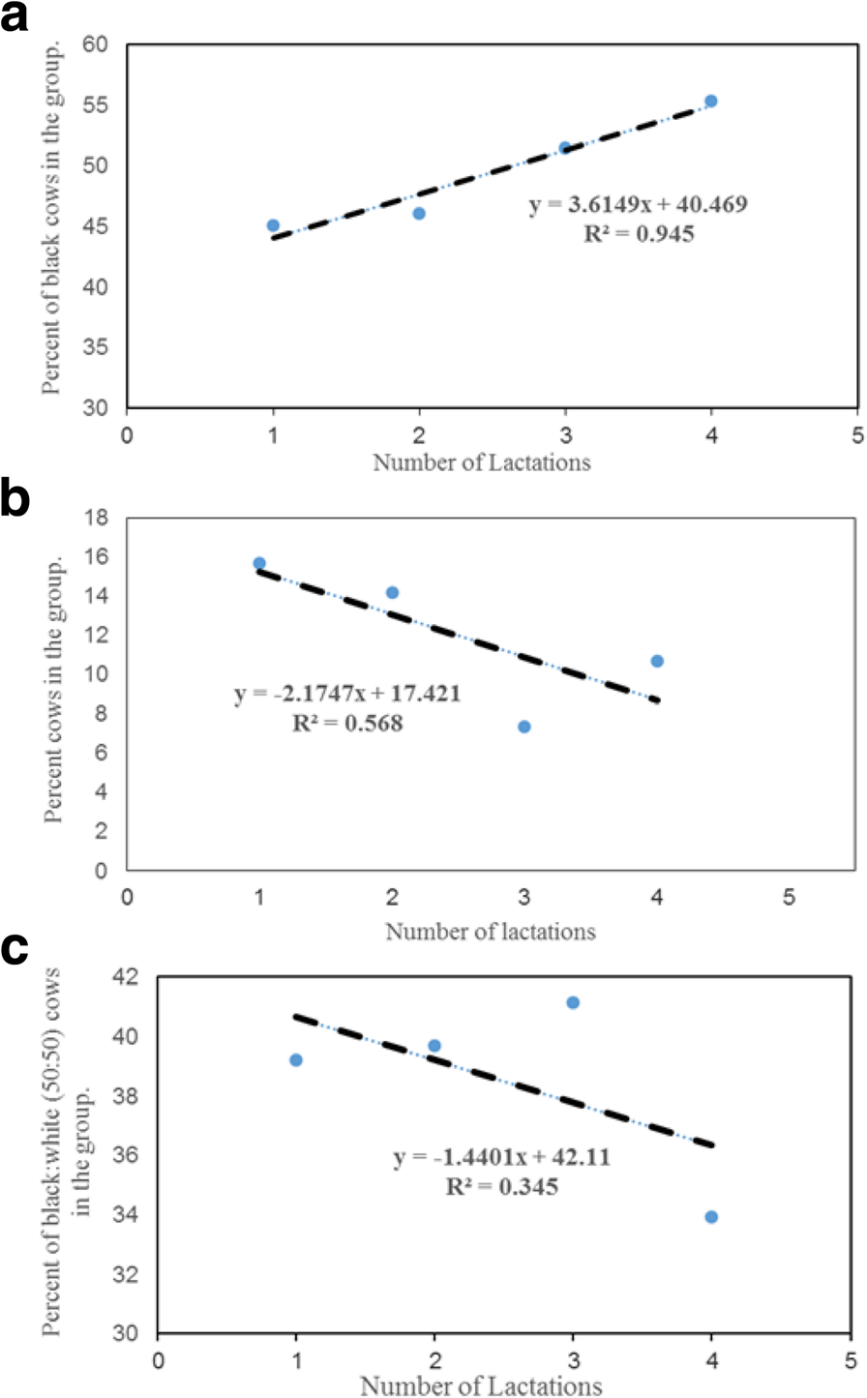
**a** Regression analyses of black cows with 2 to 5 or more lactations in Farm B. **b** Regression analyses of white hair coat cows with 2 or more lactations in Farm B. **c** Regression analyses of black:white (50:50) hair coat cows with 2 or more lactations in Farm B

Figure 1a-1 and b show the regression analyses on the distribution of black and white cows (%) in relationship to the corresponding lactation for Farm A. The data showed that as the number of lactation increased, the percentage of black cow represented in the herd increased. The population of black cows went from 51.6% with 2 lactations to a high of 78.6% for cows with 6 lactations. The cows with black hair coat stood at 56.3% for the cows with 7 lactations or more. When the regression analyses were performed for 4–7 or more lactations, the *r*-square value was 0.078. This suggested that the percentage of black animals did not change in the population for this herd. However, if the regression analyses was performed for cows with 4–6 lactations, the R^2^ = 0.99; suggesting that percentage of black hair coat animals increased with lactation numbers (Figure 1a-2). This implied that black cows had greater longevity in the farm.

In commercial Holstein dairy operations, there are very few cows that made it beyond 6 lactations due to genetic gains from the replacement and other factors [22]. A cow at her 6^th^ lactations would mean she is over 9 years of age. The average productive life of Holstein cows is 2.5 lactations [28, 29].

The white cow population was 17.5% for animals with 4 lactations and this group decreased to 6.3% by the 7+ lactations (Fig. 1b). The lower population of animals with 7 or more lactation is to be expected due to genetic progress and aging of animals.

The distribution of the mixed color hair coat (black:white, 50:50) was relatively stable for the period examined (Fig. 1c; R^2^ = 0.017)). There was 30.9% of this population with 4 lactations and by 7+ lactation, this population was 37.5% of the group.

Similar findings were observed for Farm B. In this farm the population distribution of black hair coat cows increased from 45.1% in cows with two lactations to 55.4% in the group of animals with 5 or more lactations (Fig. 2a; R^2^ = 0.945). Like farm A, the mixed color population distribution was stable (39.2–33.9%, Fig. 2b; R^2^ = 0.341) and the white cow populations decreased (15.7–10.7%) for the period examined (lactations 2–5, Fig. 2c; R^2^ = 0.574). For Farm B, we chose 2–5 or more lactation for analyses because in this herd the number of animals beyond 5 lactations in the herd dropped drastically.

### Analyses of survival by Wilcoxon test for homogeneity

**Fig. 3:**
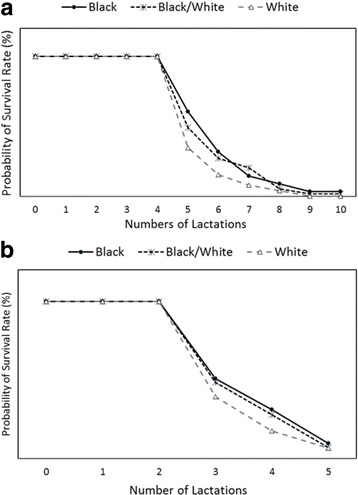
**a** Wilcoxon test for homogeneity of data for survival rate of cows by hair coat color in Farm A. Black cows had greater survival rate compared to white cows (*P* < 0.05). **b** Wilcoxon-test for homogeneity of data for survival rate of cows by hair coat color in Farm B. Black cows tend to have better survival rate than white cows (*P* < 0.06)

The data were further subjected to Wilcoxon-Log-rank test for survival probability with respect to lactation numbers. The Wilcoxon test placed more weight on the smaller number of lactation while the Log-rank test placed more weight on the larger lactation numbers. The results show that for both farms, black cows had a better survival rate (Fig. 3a and b). Table 3 showed that mean median lactation survival time by hair coat for the respective farms. In farm A, black hair coat cows lived 7.2 months longer than white hair cows and in farm B, they had 2.4 months longer survival rate.

**Table 3:** Median lactation survival time (year) for cows by hair coat color (mean ± SE)

Farm A		Farm B	
Hair coat	Years	Hair coat	Years
Black	5.06 + 0.16	Black	3.04 ± 0.14
Black/White	4.98 + 0.13	Black/White	2.97 ± 0.06
White	4.76 + 0.15	White	2.82 ± 0.09

In the USA, the average productive life of a dairy cow is 2 years. Eight months [16]. In most cases, this equates to 2.45 lactations [28, 29]. Guernseys leave the herd fastest followed by Holsteins. Jerseys have the longest productive life. Longevity in dairy herds could potentially mean higher lifetime milk yield, more calves born, greater adaptability to the mirco-environment, greater contribution to genetic progress of a herd due to the availability of replacements and better adaptability to local management. All these factors lead to lower cost of production and greater profitability for a dairy operation.

Table 4, showed the measurements of hair coat parameters obtained in this study. The weight of white hair was heavier than black hair, lending it to higher μg/cm^2^ (18.4 vs 8.2 μg/cm^2^). It was observed that the white hair was thicker and longer while the black hair were shorter and smaller in diameter. The length, thickness and hair count per cm^2^ was performed in subsequent study (not yet published). Gebremedhin et al. [11] showed that hair coat can be a barrier for evaporative cooling. The removal of hair enhanced sweating rates. The white hair coat on unshaved cows had only 87% in sweating rate efficiency compared to black unshaved cows.

**Table 4:** Weight of hair follicles (μg/cm2) obtained on the 11^th^ rib region of the cow’s body

Hair color	No. of samples	μg/cm2 (mean ± SE)
Black	11	8.2^a^ ± 0.59
White	11	18.4 ± 1.28

Different superscript within a column denotes *P* < 0.05

Evidence of a major gene influencing hair length and heat tolerance was reported by Olson et al. [23] for *Bos taurus* cattle. The gene influencing hair length has been mapped on chromosome 20 in Senepol derived cattle [21]. In cross-bred Holstein animals with this gene, the hair coat is short and shiny and the sweating rates are higher than their contemporaries and they had higher milk production Olson et al. [24].

**Fig. 4:**
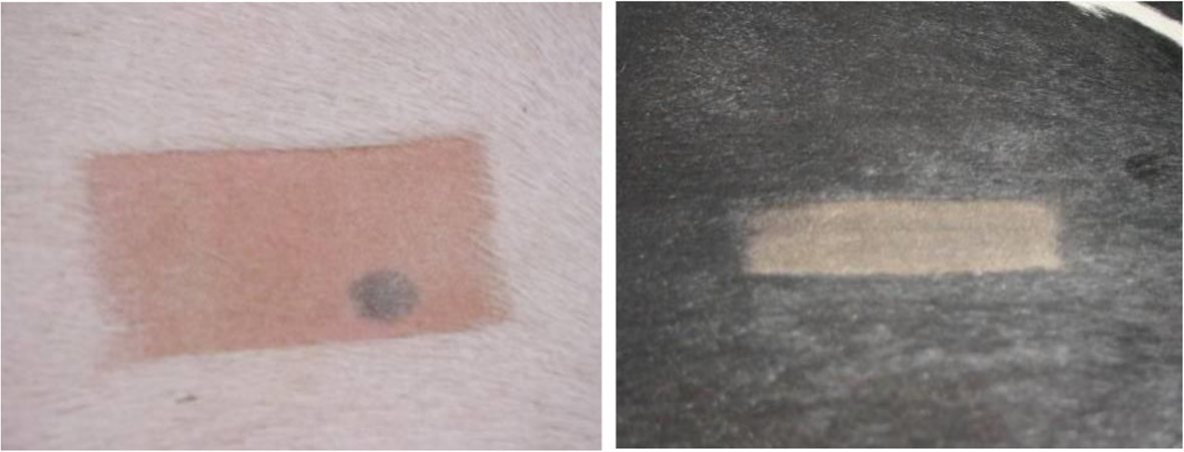
The skin color of underneath the white or black hair coat of a Holstein cow. Grey spots under white hair coat are sometimes seen in white Holstein cows. This picture showed that underneath the white hair coat, the skin is pink

**Fig. 5:**
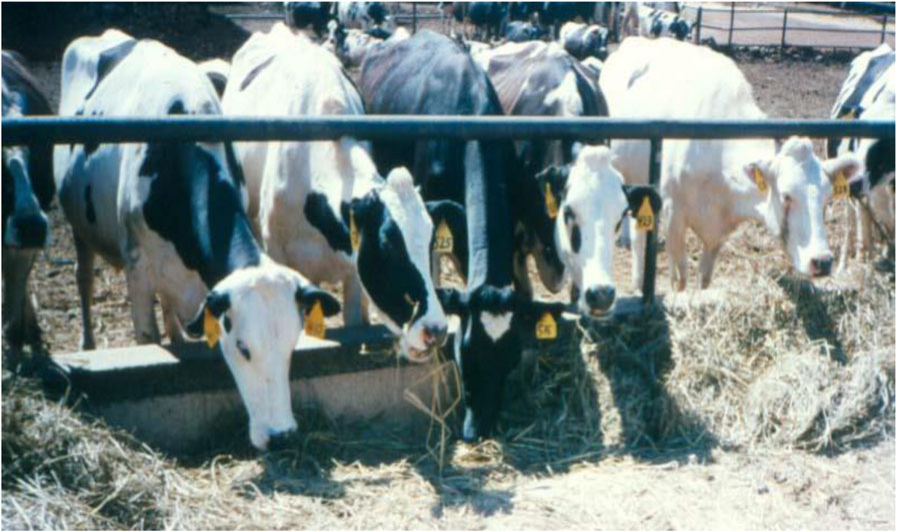
The different hair coats of Holstein cows; notice the stain on the face of the white cow due to constant tearing or irritation from solar reflection when there is no pigment in the eye lid. Tearing is absence in the 1^st^ and 4^th^ cow in the picture with pigment on and around the eye

Figure 4 shows the underlying skin color of white hair coat Holstein is pink and that for black hair coat Holstein to be grey or black. This picture also showed a black spot in the white color. Sometimes in white hair coat Holsteins, spots can be observed. It is more common in the thurl portion of the animal. The hair above this spot is white. Finch et al. [6] had eluded that there may be other reasons why Bedouins have selected black goats over white. Photo-chemical damage was one possible reason given. Hansen and Arechiga [15] alluded to the potential of skin damage due to solar radiation. Personal travels in South East Asia, as director of marketing for a genetic company in the 1990s, enforced this concept as many small holder dairy producers would avoid the purchase of semen from predominantly white Holstein sires. The effect of intense solar load in the tropics on eye cancer on cattle breeds has been reported [9]. The lack of pigmentation on the eyelids was observed in the white cow population and the faces of these animals were stained by constant tearing (Fig. 5). Sunlight or ultra violet light exposure is seen as a causative irritant for the eyes in cattle without pigmentation around the eyelids [31]. This lack of pigmentation has been reported to be detrimental in cattle [1] and this is highly heritable in cattle [32]. This may be one possible reason for the lower number of white hair coat cows found in both farms as the lactation number increased.

## Conclusion

In conclusion the study shed light on the relationship of hair coat color and survival rates for Holstein in the hot climates. Along with the pool of evidence in the literature, cows that have longevity in the tropics must have greater ability for evaporative cooling; and this was probably coupled with darker skin color (pigmentation), hair coat color and the physical characteristics of the hair. Further investigation in the relationship of thermal regulations to hair and skin color and characteristics are important in the presence of climate change.

## Abbreviations

B: 90% black
BW: Black:White (50:50)
kg: Kilogram
W: 90% white
μg: Microgram
